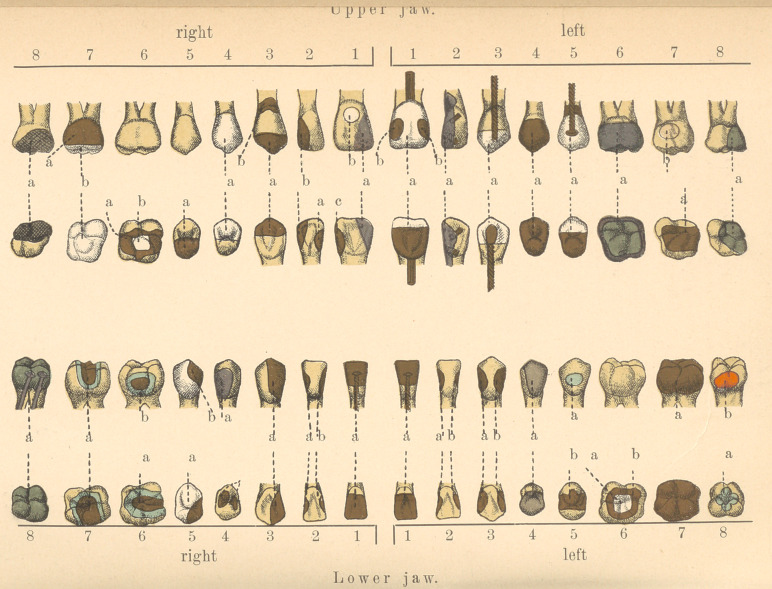# Various Modes Adopted in Filling Teeth

**Published:** 1890-10

**Authors:** Wilhelm Sachs

**Affiliations:** Breslau, Germany


### VARIOUS MODES ADOPTED IN FILLING TEETH.[1]

BY WILHELM SACHS, D.D.S., BRESLAU, GERMANY.

I HAVE the honor to show you here a skull with a full set of natural teeth, which I have prepared and filled with the different materials used in dental practice. The various forms of the cavities and fillings are such as we generally meet with. If you follow my explanations, as illustrated in these engravings, I hope you will understand my description of the work more clearly. We commence with (1) the upper right central incisor. This contains on the mesial surface a large platinum-gold contour filling. This material is probably not known to all the gentlemen present, therefore I would mention that it is composed of a layer of thin platinum foil covered with gold, having the appearance of the common gold foil. When it is put into the tooth cavity, trimmed, and polished, it has the shade of nearly pure platinum with a very slight gold color. Many dentists believe that it is less objectionable in regard to the appearance than a pure gold filling, and is claimed to be harder and more resistant than the latter. Concerning the working of platinum-gold, I would state that it is annealed like cohesive gold foil, to which it also adheres. It can be worked with hand-pressure and mallet. While annealing, care must be taken not to burn the gold, leaving the platinum foil uncovered. In the same tooth you see on the labial surface a porcelain inlay, which is, in regard to appearance, the most tooth-like material we can use. This inlay is made as follows: With a wheel-shaped bur, after

---

[1] Read at the meeting of the American Dental Society of Europe, August 6, 1889.

Upper jaw.
right
left
8 7 6 5 4 3 2 1
1 2 3 4 5 6 7 8
8 7 6 5 4 3 2 1
1 2 3 4 5 6 7 8
right
left
Lower jaw.

the excavation, the cavity is formed perfectly round and as deep as possible, taking care not to expose the pulp. The walls must be at a right angle to the bottom, with a slight undercut. Then an artificial tooth of the proper color is ground nearly to the size of the cavity, leaving it a little larger. Only American teeth are fit for this purpose; English teeth show, after the grinding and fastening, a different shade of a gray, greenish character. The White Manufacturing Company have now for this purpose small round porcelain pieces of different sizes and shades. These have the great advantage that the material is dense and permits, after grinding, a perfect polish of the exposed surface. The piece of porcelain is fastened, by shellac, on the point of an old worn-out engine-bur, and this is put in the hand-piece of the engine while the engine is turned to the right; the porcelain is held against a fine corundum wheel of the laboratory lathe, which is turned to the left. The inlay is ground slightly conical, to fit exactly the entrance of the tooth cavity. If it happens to be a little too small, grind off the portion from the pointed end, and it will exactly touch the walls of the cavity at all points. If the size of the inlay permits of it, a shallow groove may be cut, with a sharp Arthur disk, into the side of the inlay, where the cement, which is used for the fastening, can enter. After trying whether the piece will fit the cavity, you can fill this with fine pumice, and grind with the engine the inlay into the cavity; in this manner you get the most perfect fit imaginable. Then the porcelain is taken off from the pin, cleaned with alcohol, and set with soft mixed cement, which must get entirely hard before you grind and polish the exposed surface with corundum and a Kansas stone.

It is recommended to fasten with Hill's stopping, but I have found this to cause a dark and unsightly rim. If such inlays are made correctly it requires a close attention to detect them. Such work will always give great satisfaction to patient and operator. In regard to durability, it will last, if properly done, many years. There are yet several methods for making porcelain inlays, but I fear it would take up too much of your time. I mention these, as I have yet several things to speak of that will be, perhaps, of some interest to you.

Fillings, such as *c* in the upper right central and *a* in the upper right lateral incisor, we often have to make. I call it a professional crime to open such cavities from the labial surface, if we can prevent it. It is not only our duty to preserve teeth, but also to preserve the good appearance of them, as far as it may be in our power. To work at such places from the palatine surface a high skill in the use of the mouth-mirror is required. The mirror should be of a large size, round form, and only very little magnifying. A glass of higher magnifying power will distort the object reflected. Filling *c* in the central tooth is a contour gold filling, held by undercuts, retaining-points, and holes; this I do not recommend. The canine has two gold fillings, *a*, which restore the lost cutting edge; *c* is a filling in a cervical cavity. To keep this free from moisture while filling, the use of the new How's cervical clamp is recommended. Lately, Dr. George Elliott, of London, has constructed a clamp for the same purpose. Not having tried it, I am not prepared to give an opinion of its value; but, to judge by the drawing, it must work satisfactorily.

(4) The labial and a part of the masticating surface consist of a porcelain crown. We often find this portion of a bicuspid broken off. If we fill the defect with gold, it will be objectionable for some patients, on account of the appearance. Cement does not allow a contour of durability, but a porcelain piece will do good service in such a place. A common, plain, half bicuspid crown is ground to fit the cavity. The pulp-chamber is enlarged for the reception of the platinum pins. The piece is set in with cement.

(5) The palatine and a part of the masticating surface of the second bicuspid is built up with gold foil.

(6) The masticating surface of the first molar holds a gold filling with a piece of porcelain ground in. The porcelain piece is practically in such place worthless; but it has a good appearance and will satisfy our patients. After the cavity is cleaned and shaped, an artificial molar is ground into it a little smaller than the cavity, and fastened with quick hardening cement. The small rim of cement between tooth and porcelain is cut out with excavators and filled with gold.

(7) The root of the second molar holds an artificial crown consisting of a gold band and a porcelain masticating surface. After grinding and shaping the root, fit a twenty-two-carat gold band exactly to the contour of the root. After soldering, push the band a little below the margin of the gum and take an impression. The gold ring, which will remain on the tooth, is taken off and placed into the impression. Take an impression of antagonizing teeth. Make the model, place them in the articulator, and grind a porcelain masticating surface, which is manufactured for this purpose, to fit the band and the articulation. The band and porcelain is fastened with cement.

(8) The wisdom tooth has a filling consisting of a mixture of amalgam and oxyphosphate of zinc cement. This material has the advantage of cement and of amalgam without their objections. In cases where deep undercuts, required for amalgam, cannot be made, and cement, on account of its very limited durability, cannot be recommended, these two materials will do good service. First prepare the amalgam in the usual manner, then mix it with the fluid and the phosphate cement powder, and introduce into the dry cavity before it gets hard. It will not take such a high polish as a pure amalgam filling, but it will last nearly as long and does not discolor the tooth.

I come now to the explanation of the left upper jaw. The central incisor of this side is a pivot tooth. The method is perhaps more difficult and complicated, and requires more skill than some others; but I claim for it that it is better, cleaner, and stronger than any kind of pivot-teeth I know. In the root is a tube with six or eight longitudinal grooves. The tube is fastened with cement, and the exposed surface of the root is covered with gold. The pivot of the artificial crown corresponds exactly with the tube and prevents the turning of the tooth in the root. I have described this pivot tooth in the *Oesterreich Vierteljahrschrift für Zahnheilkunde*, and it is reprinted in the last Poulson's *Vierteljahrschrift.* Here is a preparation which will easily demonstrate the manner in which it is done The porcelain front of the tooth contains two gold fillings. The cavities for these are ground in with diamond points. The method often recommended, to drill holes in artificial teeth with emery, turpentine, and spirit of camphor, by means of a copper drill, has been successful in my hands.

(2) The lateral incisor has a large platinum-gold filling. The cavity did not permit undercut or retaining-points, therefore I inserted two anchor screws for retaining the filling.

(3) The half of the canine is a porcelain piece. A gold pin is screwed into the root and a piece of an old-fashioned tube tooth placed over the projecting pin. The piece is fastened with cement and the end of the canal closed with a small gold filling. It is not often that we meet with such defects, but if we find a chance to perform this operation it will give us and the patient great satisfaction.

(4) The first bicuspid is almost entirely built up with cohesive gold. It was worked with the mechanical mallet and the Kirby pneumatic mallet, both driven by the electric motor.

(5) The root of the second bicuspid is attached to a Richmond crown with a buccal porcelain face. A gold band was exactly fitted to the contour of the root, then the buccal wall cut out, into which the porcelain canine is placed and soldered.

For greater security a headed gold pin is screwed into the root. The crown is fastened with Brinkmann's cement.

(6) The crown of the first molar is also an artificial substitute. It consists of a platinum band filled with amalgam. It does not look as good as a gold crown, but it is quickly made, is less expensive, and gives the same service.

(7) The masticating surface of the second molar is filled with gold extending over the distal surface up to the cervix. The approximal surface is filled with copper amalgam, a method which I very often make use of, believing that no other filling material (except gutta-percha) will prevent secondary decay so effectually. I first fill the prepared cavity entirely with copper amalgam, using the matrix. I then allow the amalgam to harden at least two days, having found that such a long time is necessary for the mercury to amalgamate perfectly with the metal. Upon this, after a portion of the amalgam on the masticating surface is removed and undercuts made, I fill with gold and finish. This combination, making the apparently difficult operation very easy, saves time and has for the conservation of the tooth at least the same effect as a pure gold filling. The buccal cavity is filled with Hill's stopping, which, in this peculiar place, is of great value.

(8) The distal wall of the wisdom tooth, including a part of the masticating surface, is filled with copper amalgam, which in such cases is the most reliable filling.

In the lower jaw you notice the crown of the right central incisor about two-thirds built up with gold foil. This extensive contour filling is held by means of an anchor screw fastened into the root-canal.

(2) The lateral incisor has two cavities, often met with in daily practice. I said before that I think it to be a professional crime to open approximal cavities from the labial surface.

That remark refers only to the upper front teeth. If we would undertake to excavate and fill the approximal cavities of lower front teeth from the lingual surface, we would transform from a relatively simple operation into a very complicated and difficult one. If time permits, in such cases, I place between the teeth common cotton for two or three days to separate them, not using sandarach varnish, which prevents the swelling of the cotton.

(3) The cutting edge of the incisor is built up with gold, an operation not very difficult, but requires much time and patience.

(4) Two small gold fillings are inserted into the masticating surface of the first bicuspid. In this case the wall between both was sufficiently strong not to require cutting away. In other cases it would be better to combine the cavities.

The buccal surface has a platinum-gold filling.

(5) The root of the second bicuspid serves as a holder of a Logan crown, which is set with cement. To get a very secure hold of the crown two or three little cross grooves can be cut into the walls of the root-canal. Also barbed hooks can be cut with a sharp knife into the edges of the square pin, the points of the barbs towards the porcelain crown.

(6) The first molar has on the masticating surface a large tin-gold filling with gold centre.

(7) The combined masticating and buccal surface of the second molar is filled with gold, but showing a free tin-gold rim covering the walls of the cavity. In regard to this material, I think it has not its equal in the proper place. It can be worked nearly as fast as amalgam, will last as well, often better, than pure gold, and will prevent secondary decay, and is as reliable as gutta-percha or copper amalgam, not staining the tooth. To those who are not familiar with this material I would advise the reading of Professor Miller's essay on this subject.

(8) The wisdom-tooth crown is built up with amalgam which is held by means of two platinum pins, headed and screwed into the root-canals. In this case a ring matrix was employed, which was left in place for twenty-four hours, until the amalgam had hardened.

(5) The buccal surface of the second bicuspid is filled with tin-gold, the masticating surface with gold, continuing on to the approximal surface down to the neck of the tooth.

(6) The first molar has upon its masticating surface a piece of porcelain, ground in, fastened with cement and surrounded by a gold rim.

(8) The root of the second molar holds a Richmond crown. There are several methods of making this; but I have only found two modes which have proved satisfactory. After treating and filling the root-canals, if this should be necessary, grind the left portion of the crown so that the walls are parallel. Then shape and fit a gold band around the root and solder to this a masticating surface, into which the cusps are swedged with a stamp, which can easily be shaped according to the case. The other method is somewhat different. Take the impression of the stump upon which you intend to make the crown. Cut out a piece of gold plate resembling in shape the German iron cross. Stamp with a punch the masticating contour and bend the side parts down to fit the root. Tie a thin wire around it, and place over the root in the mouth, and twist the ends of the wire firmly together until the gold plate has taken the exact shape of the contour of the root. Remove the crown, give one turn to the wire to get a slight fitting of the crown, and solder. Drill a hole into the masticating surface through which the surplus cement, with which the crown is fastened to the root, can escape.

(8) Filling *a* in the wisdom tooth is a cross-shaped tin-gold filling; *b*, a red gutta-percha filling. This material is not sufficiently estimated. It has the same conservative properties as Hill's stopping, but does not wear off so quickly. It should be carefully heated and pressed into the cavity with cold instruments. Overhanging portions should not be removed with warm instruments, as these would draw the gutta-percha away from the walls. After cooling off, cut the surplus away with a thin, sharp knife. English red gutta-percha is not fit for such fillings; only the red base-plate gutta-percha should be made use of.

## Figures and Tables

**Figure f1:**